# Bayesian modeling of quantiles of body mass index among under-five children in Ethiopia

**DOI:** 10.1186/s12889-024-18602-x

**Published:** 2024-04-24

**Authors:** Daniel M. Mekuriaw, Aweke A. Mitku, Melkamu A. Zeru

**Affiliations:** 1 Department of Statistics, College of Science, Bahir Dar University, Bahir Dar, Ethiopia; 2 School of Mathematics, Statistics and Computer Science, College of Agriculture Engineering and Science, University of KwaZulu-Natal, Durban, South Africa

**Keywords:** Bayesian quantile regression, BMI, Under-five children, Ethiopia

## Abstract

**Background:**

Body Mass Index (BMI) is a measurement of nutritional status, which is a vital pre-condition for good health. The prevalence of childhood malnutrition and the potential long-term health risks associated with obesity in Ethiopia have recently increased globally. The main objective of this study was to investigate the factors associated with the quantiles of under-five children’s BMI in Ethiopia.

**Methods:**

Data on 5,323 children, aged between 0-59 months from March 21, 2019, to June 28, 2019, were obtained from the Ethiopian Mini Demographic Health Survey (EMDHS, 2019), based on the standards set by the World Health Organization. The study used a Bayesian quantile regression model to investigate the association of factors with the quantiles of under-five children’s body mass index. Markov Chain Monte Carlo (MCMC) with Gibbs sampling was used to estimate the country-specific marginal posterior distribution estimates of model parameters, using the Brq R package.

**Results:**

Out of a total of 5323 children included in this study, 5.09% were underweight (less than 12.92 BMI), 10.05% were overweight (BMI: 17.06 – 18.27), and 5.02% were obese (greater than or equal to 18.27 BMI) children’s. The result of the Bayesian quantile regression model, including marginal posterior credible intervals (CIs), showed that for the prediction of the 0.05 quantile of BMI, the current age of children [$\upbeta$= -0.007, 95% CI :(-0.01, -0.004)], the region Afar [$\upbeta$ = - 0.32, 95% CI: (-0.57, -0.08)] and Somalia[$\upbeta$ = -0.72, 95% CI: (-0.96, -0.49)] were negatively associated with body mass index while maternal age [$\upbeta$ = 0.01, 95% CI: (0.005, 0.02)], mothers primary education [$\upbeta$= 0.19, 95% CI: (0.08, 0.29)], secondary and above [$\upbeta$ = 0.44, 95% CI: (0.29, 0.58)], and family follows protestant [$\upbeta$ = 0.22, 95% CI: (0.07, 0.37)] were positively associated with body mass index. In the prediction of the 0.95 (or 0.85?) quantile of BMI, in the upper quantile, still breastfeeding [$\upbeta$ = -0.25, 95% CI: (-0.41, -0.10)], being female [$\upbeta$ = -0.13, 95% CI: (-0.23, -0.03)] were negatively related while wealth index [$\upbeta$ = 0.436, 95% CI: (0.25, 0.62)] was positively associated with under-five children’s BMI.

**Conclusions:**

In conclusion, the research findings indicate that the percentage of lower and higher BMI for under-five children in Ethiopia is high. Factors such as the current age of children, sex of children, maternal age, religion of the family, region and wealth index were found to have a significant impact on the BMI of under-five children both at lower and upper quantile levels. Thus, these findings highlight the need for administrators and policymakers to devise and implement strategies aimed at enhancing the normal or healthy weight status among under-five children in Ethiopia.

## Background

Health is a positive, multifaceted concept that can encompass a multitude of elements, including capability, judgment, enjoyment, and well-being. The Body Mass Index (BMI) is a metric used to assess nutritional status. Additionally, BMI is used to evaluate a person's weight status in both adults and children. However, while BMI cut points for obesity and overweight are the same for both sexes and age groups in adults, they alter for growing children based on their age and gender [1].

The BMI of a person can be used as a screening tool to determine whether or not they are obese, overweight, underweight, or at a healthy weight for their height. The BMI is a weight measurement that takes height into consideration. It is calculated by dividing weight in kilograms by height in meters squared (kg/m^2^) [2, 3]. Obese, overweight, and normal (healthy weight) were defined as children's BMIs for under-five children that were at or above the 95^th^ percentile, between the 85^th^ and 95^th^ percentile, and between the 5^th^ and 85^th^ percentile, respectively [4]. For children, BMI is dependent on age and sex and is often referred to as BMI-for-age. A person's risk of disease or death may rise dramatically if their BMI is higher than the acceptable limit [5]. Both being underweight and having a large amount of body fat increase the risk of developing disorders linked to weight and other health problems in adults and children [6–8]. BMI is significantly associated with relative fatness in childhood and adolescence and is the most convenient way of measuring relative adiposity [9].

Particularly in Ethiopia, a nation with a low income where childhood malnutrition is still a major problem, pediatric obesity (BMI above the 95^th^ percentile) is not yet seen as a serious health concern and is given little attention. The prevalence of overweight (BMI between the 85^th^ and 95^th^ percentile) children in Ethiopia has increased overall, from 1.7 to 3.6%, according to the United Nations Children's Fund (UNICEF 2017) annual report [10]. Despite the high prevalence of childhood malnutrition in Ethiopia, there is limited understanding of the factors influencing the distribution of body mass index faced by specific groups of under-five children [11].

Being overweight and/or obese during puberty increases the risk of contracting non-communicable diseases and contributes to overweight, obesity, cardiovascular disease, metabolic and other diseases in adulthood. Therefore, primary prevention requires information about the lower, and upper-level, classification and underlying factors of BMI in developing countries. Consequently, new insights into the data sets can be obtained by applying quantile regression as an alternative to the conventional techniques of linear or logistic regression models [12, 13]. However, the interest lies in the lower and upper spectrum of BMI, these regression models are based on mean BMI. Quantile regression, a natural extension of classical mean regression is a method that is used to model a relationship between the quantile of variable response and one or more variable predictors [14].

Quintile regression seems to provide a better fit than traditional generalized linear models (GLMs) for estimating risk factors based on BMI data. Quantile regression is recommended in situations where the data are heterogeneous, meaning that the centers and tails of the conditional distributions fluctuate differentially with the covariates [15]. Quantile regression offers a thorough understanding of the interactions between independent and dependent variables (i.e., not just in the center but also in the tails of the dependent variable's conditional distribution) [16].

Quantile regression models (QRM) the impact of predictors on different specific quantiles (or percentiles) of the response distribution, and thus provide a more comprehensive picture of the effect of predictor variables on the spectrum of the response variable [17, 18]. An additional advantage of the quantile regression approach is that its parameter estimates are not affected by changes in the conditional distribution of the dependent variable, which is the BMI of the children, on a location-scale [19]. In the health sciences, quantile regression has become popular concerning studies of BMI [12, 20, 21].

Bayesian methods provide parameter estimates with good statistical properties, parsimonious descriptions of observed data, predictions for missing data and forecasts of future data, and a computational framework for model estimation, selection, and validation [22]. Bayesian techniques use prior distribution to describe sample data and population characteristics. The posterior distribution can be obtained by combining sample data with the prior distribution on the model parameters. In order to estimate a quantile regression parameter using the Bayesian technique, one must ascertain the posterior distribution, which is proportional to the sum of the likelihood function and the prior distribution The computation of posterior distribution can be difficult and time-consuming to calculate analytically if more parameters are to be estimated. Therefore, estimating parameters has been used as a computational method.

Since the mean regression only provides for the description of the distribution's mean response, BMI employing the Bayesian technique quantile regression is more pertinent due to its flexibility in estimating conditional quantiles of interest of a given distribution. In order to model big data sets, we employed quantile regression techniques and an estimation of Bayesian methodologies for this work [23, 24]. So far, there have not been many detailed studies conducted to explore all aspects of BMI in Ethiopia using a quantile regression model rather they only focused on fixed effects. The current study adopted a Bayesian quantile regression model to analyze the BMI of under-five children by including the regional variation.

## Data and methods

The section emphasizes the study population, data sources, data analysis approaches, and proposed quantile estimation approach.

### Data and sampling procedure

The data was secondary data obtained from the Ethiopia Mini Demographic and Health Survey (mini EDHS) (2019). The 2019 mini EDHS) was implemented by the Ethiopian Public Health Institute, in partnership with the Central Statistical Agency and the Federal Ministry of Health, under the overall guidance of the Technical Working Group. Data collection took place from March 21, 2019, to June 28, 2019. The data are openly available from https://dhsprogram.com and can be accessed following the protocols. To incorporate the geographical covariates, most of the data usually includes global positioning system coordinates [25].

The Ethiopian Demographic and Health Survey used a two-stage stratified cluster sampling technique selected from a population and housing census frame for the 2019 mini EDHS. In the first stage, a total of 305 Enumeration Area EAs (93 in urban areas and 212 in rural areas) were selected with probability proportional to EA size and with independent selection in each sampling stratum. In the second stage of selection, a fixed number of 30 households per cluster were selected with an equal probability of systematic selection from the newly created household listing. A total of 9,150 households were selected for the sample, of which 8,794 were occupied. Of the occupied households, 8,663(99% response rate) were successfully interviewed. The women were interviewed by distributing questionnaires and information on their birth history and 5,323 under-five children were considered for this study [26].

### Variables

Variables considered in the study were based on some previous studies and those that are expected to be factors or determinants of under-five age of children BMI. We have considered under-five children’s BMI as the response variable. BMI (in a standardized form) was used as a continuous variable and computed as:

$${\mathrm{child^{\prime}}{\text{s}}}_{{\text{BMI}}}=\frac{\mathrm{child^{\prime}}\mathrm{s\;weight }\;(\mathrm{in\;kilogram}) }{{(\mathrm{child^{\prime}}\mathrm{s\;height }\;(\mathrm{in\;meters}))}^{2}}$$

The covariates were the variables that are expected to affect the response variable. From many kinds of literature, the following are those that affect the BMI of under-five children (Table 1).

**Table 1:** Description of the independent variable

Socio-Demographic Level Covariate	**Description**
Sex	Sex of children (Male ”M” or Female “F”)
Age	Current Age of children (0 – 59) month’s
Mother’s Age (MA)	Respondents' Current age 15 - 49 years
Household Size(HS)	Number of Households listed (<4, 5-9, 10+ )
Number of UnderFive age Children (NUFC)	Number of children under the age of 5 (<2, 2, & 3 or more)
Type of Birth (TB)	multiple Vs Singleton
Socio-economic Level covariate	
Place of Residence (PA)	Rural Vs Urban
Mother’s Educational level (ME)	No formal education, Primary and Secondary and above
Marital Status	Married Vs not married
Wealth Index(WI)	Poorest, Poor, Middle, Richer, and Richest
Region(Reg)	Tigray, Afar, Amhara, Oromia, Somali, Benishangul-Gumuz, SNNPR, Gambela, Harari, Addis Ababa, Dire Dawa
Behavioral Level covariate	
Religion(Rel)	Orthodox, Muslim, Protestant, and others (catholic, tradition, and other)
Breastfeeding (BF)	Duration of Breastfeeding (still breastfeeding, never breastfed and ever breastfed and not currently breastfed)
Water Source and Sanitation Level Covariate	
Source of Drinking Water (SDW)	Improved and Unimproved
Toilet Facility (TF)	Improved and Unimproved

### Statistical methods

#### Quantile regression

Quantile regression is a regression method that models a relationship between the quantile of variable response and one or more variable predictors. Quantile regression is robust to outliers and can model data with a heteroscedasticity effect because it offers the opportunity for a more complete view of the response variable and the relationships among predictor variables. The QRM estimates the potential differential effect of a covariate on various quantiles in the conditional distribution, therefore, we are interested in estimating quantiles of the response distribution as a function of potential Predictor variables. When the conditional densities of the response are heterogeneous, it is natural to consider whether weighted quantile regression might lead to efficiency improvements [14, 17, 18]. An alternative method for dealing with outliers is quantile regression. Quantile is defined as a particular location of some distribution, where τ^th^ quantile is the value of y when ${P}_{r}\left({\text{Y}}\le {\text{y}}\right)=\uptau$ where τ has a value between 0 and 1.

A useful property of the conditional quantile function is its invariance to any monotone transformation of the response variable that is for any monotone function h(**.**), We have ${Q}_{{\text{h}}}(\mathrm{ Y })|{\text{X}}(\uptau ) =\mathrm{ h}({Q}_{{\text{Y}}} |{\text{X}}(\uptau ))$.

The quantile regression model is described by the conditional *τ *^*th*^ quantiles of the response *Y* for given values of predictors ${x}_{1},{x}_{2},\dots ,{x}_{k}$. The linear quantile regression model for a set of covariates,

X, is given by

1 $${{\varvec{Y}} ={{\varvec{X}}^{\prime}}_{{\varvec{i}}}{\varvec{\beta}}(\tau ) + {u}_{i}}$$

where ${{\varvec{X}}}_{{\varvec{i}}}$ **is** a set of covariates, the ${u}_{i}$ , is a vector of independent errors which are independent and satisfy $P({u}_{i}<0|{{\varvec{X}}}_{{\varvec{i}}})=\tau$. It is a natural extension of the traditional mean model.

2 $${\text{Qy}}\left(\uptau |{x}_{1},{x}_{2},\dots ,{x}_{k}\right)={{\varvec{\upbeta}}\left(\uptau \right)}_{0}+{{\varvec{\upbeta}}\left(\uptau \right)}_{1}{{\text{x}}}_{1}+... +{{\varvec{\upbeta}} (\uptau )}_{{\varvec{k}}}{{\text{x}}}_{k}, 0<\tau < 1$$

where ${{\varvec{\upbeta}}({\varvec{\uptau}}) = (\upbeta \left(\uptau \right)}_{0},{\upbeta \left(\uptau \right)}_{1},... ,{\upbeta (\uptau )}_{k}$) is the unknown parameter vector.

Equation (2) gives the changes in the conditional quantiles. Because any *τ *^*th*^ quantile can be used, any predetermined situation of the distribution can be modeled [27]. This is useful to obtain a more complete understanding of how the outcome distribution can be affected by the predictors.

#### Bayesian quantile regression

Bayesian quantile regression is a regression method that models a relationship between the quantile of variable response and one or more variable predictors with parameter estimation used in the Bayesian method. A Bayesian quantile regression model with “k” independent variables is:

3 $$y={\upbeta (\uptau ) }_{0}+{\upbeta (\uptau ) }_{1}{x}_{1}+\dots + {\upbeta \left(\uptau \right)}_{k}{x}_{k}+ \varepsilon$$

where y is a response variable, ${x}_{k}$ is a k^th^ predictor variable, ${\upbeta \left(\uptau \right)}_{k}$ is a k^th^ regression parameter for τ^th^ quantile, and $\varepsilon \sim \mathrm{Asymmetric\ Laplace\ Distribution }\left({\text{ALD}}\right)\uptau$ is the error term for Bayesian quantile regression. Bayesian quantile regression parameters can be estimated with sample data. Suppose that *p*>k observations are available and let y_i_ denote the i^th^ observed response, and x_ij_ denote i^th^ observation or level regressor of x_j._ Actually, n is a more standard notation for the sample size (number of observations), instead of p.

For the linear quantile regression, no specific assumptions regarding the error term are made except that given a fixed and known quantile τ ∈ (0, 1), it is assumed that the τ ^th^ quantile of the error term is zero, i.e. F^−1^(τ |$\pi$) = 0 and that ε_i_ and ε_j_ are independent for i ≠ j. With these assumptions, the quantile-specific regression coefficients β_(τ)_ are estimated by minimizing an asymmetrically weighted sum of absolute deviations.

4 $$\widehat{{\varvec{\upbeta}}}(\uptau ) = {\vphantom{\sum}}_{\beta (\tau )}^{min}{\sum }_{i=1}^{p}\rho ({{\text{y}}}_{{\text{i}}}- {\mathbf{x}\mathrm{^{\prime}}}_{{\text{i}}}{\varvec{\upbeta}}(\uptau ))$$

5 $$\widehat{{\varvec{\upbeta}}}\left(\uptau \right)= {\vphantom{\sum}}_{\beta (\tau )}^{min}\left\{\uptau {\Sigma }_{{\text{i}}:{\text{yi}}\ge \mathrm{x{\prime}}{\text{i}}}\left|{{\text{y}}}_{{\text{i}}}-{\mathbf{x}\mathrm{^{\prime}}}_{{\text{i}}}{\varvec{\upbeta}}(\uptau )\right|+\left(1-\uptau \right){\Sigma }_{{\text{i}}:{\text{yi}}\ge \mathrm{x{\prime}}{\text{i}}}\left|{{\text{y}}}_{{\text{i}}}-{\mathbf{x}\mathrm{^{\prime}}}_{{\text{i}}}{\varvec{\upbeta}}(\uptau )\right|\right\}$$

where $\rho$(w) is a loss function defined by:

$$\begin{array}{c}\rho ({\text{w}})=\{\uptau -{\text{I}}({\text{w}}<0)\}{\text{w}}\\ \rho ({\text{w}})={\{}_{\mathrm{w\tau },{\text{w}}\ge 0}^{{\text{w}}\left(\uptau -1\right),{\text{w}}<0}\end{array}$$

where I(w < 0) is the indicator function of w. However, the check function in Eqs. (4) and (5) is not differentiable at zero when y_i_ = **x**′_i_**β**(τ), resulting in the explicit solution of minimization can’t be solved analytically. Therefore, linear programming methods are commonly applied to obtain quantile regression estimates of **β**(τ) such as the simplex method, interior point, and heuristic method [28, 29].

Bayesian quantile regression by demonstrating that minimizing in Eq. (5) is equivalent to maximizing probability function based on an error distributed ALD. However, it has the same issue as minimizing in Eq. (5) since the check function is not differentiable at zero when y_i_= **x**_i_**β**(τ), hence a different technique must be used to estimate the Bayesian quantile regression parameter [30]. According to ALD can be represented as a combination of exponential and Normal distribution. It can be written as:

$${\upvarepsilon }_{\mathrm{i }}=\upgamma {l}_{i}+{{\text{h}}m}_{{\text{i}}}\sqrt{{l}_{i}}$$

Where, *l*_i_ ~ exp (1), *m*_i_ ~ ***N***(0,1), $\upgamma$ = $\frac{(1-2\uptau ) }{\uptau (1-\uptau )}$, h = $\sqrt{\frac{2 }{\uptau (1-\uptau )}}$, i = 1,..,p and *l*_i_ and *m*_i_ are mutually independent. From this result, the Bayesian quantile regression model for sample data can be rewritten as:

6 $${{\text{y}}}_{{\text{i}}}= \mathbf{X}{\varvec{\upbeta}}(\uptau ) + {\upgamma l}_{i} +{{\text{h}}m}_{{\text{i}}}\sqrt{{l}_{i}}\mathrm{ i }= 1,\dots ,{\text{p}}, {l}_{{\text{i}}}\sim \mathrm{ exp }(1), {m}_{{\text{i}}} \sim {\varvec{N}}(\mathrm{0,1})$$

The likelihood function of **y** given ***l*** is:

$$f(\mathbf{y} | {\varvec{l}},{\varvec{\upbeta}}(\uptau )) =\prod\nolimits_{i=1}^{p}\frac{1}{\sqrt{2\pi }\sqrt{{l}_{i}}h}{exp}^{\left(-{\frac{\left({\text{yi}}-\mathbf{X}{\varvec{\upbeta}}\left(\uptau \right)-\upgamma {l}_{i}\right)}{{2 h}^{2}{l}_{i}}}^{2}\right)}$$

where **y**= (y_1_, y_2_, …,y_p_)’,***l*** = (*l*_1_,*l*_2_,…, *l*p)’, and **β**(τ) and y_1_|*l*_1_,y_2_|*l*_2_,..,y_p_|*l*_p_ are independent

The prior distribution for **β**(τ) is a Multivariate Normal With **β**(τ) ~ N(**β**(τ) _0_, ${\varvec{\omega}}$(τ)_0_) and its Probability Density Function (pdf) is:

7 $${\text{P}}({\varvec{\upbeta}}(\uptau ))=\frac{1}{\sqrt{2\pi }{|{{\varvec{\omega}}\left(\uptau \right)}_{0}|}^{-\frac{1}{2}}}{e}^{-\frac{1}{2}({\varvec{\upbeta}}\left(\uptau \right)-{{\varvec{\upbeta}}(\uptau )}_{0}){\prime}{{{\varvec{\omega}}\left(\uptau \right)}_{0}}^{-1}({\varvec{\upbeta}}\left(\uptau \right)-{{\varvec{\upbeta}}(\uptau )}_{0})}{\text{exp}}\left(-\frac{1}{2}({\varvec{\upbeta}}\left(\uptau \right)-{{\varvec{\upbeta}}(\uptau )}_{0}){\prime}{{{\varvec{\omega}}\left(\uptau \right)}_{0}}^{-1}({\varvec{\upbeta}}\left(\uptau \right)-{{\varvec{\upbeta}}(\uptau )}_{0})\right)$$

where ${{\varvec{\upbeta}}(\uptau )}_{0}$ is a vector mean of **β**(τ) and ${{\varvec{\omega}}\left(\uptau \right)}_{0}$ is a covariance matrix of **β**(τ). The reason for multivariate normal usage is to simplify Gibbs sampling calculation and form posterior distribution to rationalize with likelihood function.

The posterior distribution of **β**(τ) is given by:

8 $${\text{P}}({\varvec{\upbeta}}(\uptau )|\mathbf{y},{\varvec{l}})\propto f(\mathbf{y} | {\varvec{l}},{\varvec{\upbeta}}(\uptau ))\mathrm{ p}( {\varvec{\upbeta}}(\uptau ))$$

$$\mathrm{P }({\varvec{\upbeta}}(\uptau ) | \mathbf{y}, {\varvec{l}})\propto \prod_{i=1}^{p}\frac{1}{\sqrt{2\pi }\sqrt{{l}_{i}}h}{e}^{\left(-{\frac{\left({\text{yi}}-\mathbf{X}{\varvec{\upbeta}}\left(\uptau \right)-\upgamma li\right)}{{2 h}^{2}{l}_{i}}}^{2}\right)}\times \frac{1}{\sqrt{2\pi }{|{{\varvec{\omega}}\left(\uptau \right)}_{0}|}^{-\frac{1}{2}}}{e}^{\left(-\frac{1}{2}({\varvec{\upbeta}}\left(\uptau \right)-{{\varvec{\upbeta}}(\uptau )}_{0}){\prime}{{{\varvec{\omega}}\left(\uptau \right)}_{0}}^{-1}({\varvec{\upbeta}}\left(\uptau \right)-{{\varvec{\upbeta}}(\uptau )}_{0})\right)}$$

Prior distribution of ${l}_{i}$ is used to fulfill Gibbs's sampling need and tune **β**(τ) to get good acceptance rates. Prior distribution of ${l}_{i}$ is an exponential distribution with ${l}_{i}$ ~ exp(1) and its pdf is:P(*l*_i_)=exp(*l*_i_)

The joint distribution of *l*_1_, *l*_2_,.., *l*_p_ which is a prior distribution of ***l*** is:

9 $${\text{P}}({\varvec{l}})={e}^{\left(-\sum_{i=1}^{p}{l}_{i}\right)}$$

Posterior distribution of ***l*** is:

10 $$\begin{array}{c}\mathrm{P }({\varvec{l}}| \mathbf{y}, {\varvec{\upbeta}}(\uptau ) ) \propto f (\mathbf{y} | {\varvec{l}},{\varvec{\upbeta}}(\uptau ))\mathrm{ p}( {\varvec{l}})\\ {\text{P}}({\varvec{l}}|\mathbf{y},{\varvec{\upbeta}}(\uptau ))\propto \prod_{i=1}^{p}{{l}_{i}}^{-\frac{1}{2}}{exp}^{\left( -\frac{1}{2}\{{{\delta }_{i}}^{2}{{l}_{i}}^{-1}+{{{\varphi }_{i}}^{2}l}_{i}\}\right)}\end{array}$$

where $${{\delta }_{i}}^{2}$$ = $${\frac{\left({\text{yi}}-\mathbf{X}\mathrm{i }{\varvec{\upbeta}}\left(\uptau \right)\right)}{{ h}^{2}}}^{2}$$ and $${{\varphi }_{i}}^{2}=\frac{{\upgamma }^{2}}{{h}^{2}}+2$$ [29].

Since Eq. (10) is the kernel of a generalized inverse Gaussian $(\mathcal{G}I\mathcal{G})$ distribution, we have

11 $${\varvec{l}}| \mathbf{y}, {\varvec{\upbeta}}(\uptau ) \sim \mathcal{G}I\mathcal{G}\left(\frac{1}{2},{\delta }_{i},{\varphi }_{i}\right)$$

where the pdf of $\mathcal{G}I\mathcal{G}$($v,\alpha ,b$) is given by

$f\left(x|v,\alpha ,b\right)=\frac{{\left({}^{b}\!\left/ \!{}_{a}\right.\right)}^{v}}{2{k}_{v}\left(ab\right)}{x}^{v-1}{exp}^{\left\{-\frac{1}{2}\left({a}^{2}{x}^{-1}+{b}^{2}x\right)\right\}}$, x>0, $-\infty <v<\infty$, $\alpha ,b\ge 0$ and ${k}_{v}\left(.\right)$ is a modified Bessel function of the third kind [29].

MCMC simulation using the Gibbs-sampling algorithm was employed to draw samples from the posterior from which posterior means could be obtained. The posterior inference was implemented using Gibbs sampling this algorithm implements the Bayesian quantile regression (BQR) numerical method to directly perform the computation of fully Bayesian posteriors for the complex quantile regression model. In particular, the Bayesian quantile regression models with the structure of Gibbs sampling algorithm for the quantile regression are constructed by updating β, $v,$ and σ from their full conditional posteriors [31]. The algorithm can be summarized by the following steps:

- Step1. Determine the τ or quantile of the regression model
- Step2. Determine the initial value of ${{\varvec{\upbeta}}(\uptau )}^{0}$, ${{\varvec{v}}}^{0}$ and ${\upsigma }^{0}$
- Step3. Determine the number of samples, suppose the number of samples is k

$$\begin{array}{c}{{\varvec{\upbeta}}(\uptau )}^{1}\mathrm{from P }({{\varvec{\upbeta}}(\uptau )}^{1}| \mathbf{y},{{\varvec{v}}}^{ 0},{\upsigma }^{0})\\ {{\varvec{v}}}^{1 }\mathrm{from P }({{\varvec{v}}}^{1}| \mathbf{y},{{\varvec{\upbeta}}(\uptau )}^{1},{\upsigma }^{0}),\\ {\upsigma }^{1}\mathrm{from P }({\upsigma }^{1}| \mathbf{y},{{\varvec{\upbeta}}(\uptau )}^{1},{{\varvec{v}}}^{1}),\end{array}$$

$$.$$

$$.$$

$$.$$

$$\begin{array}{c}{{\varvec{\upbeta}}(\uptau )}^{k}\mathrm{from P }({{\varvec{\upbeta}}(\uptau )}^{k}| \mathbf{y},{{\varvec{v}}}^{k-1},{\upsigma }^{k-1}),\\ {{\varvec{v}}}^{k}\mathrm{from P }( {{\varvec{v}}}^{k}| \mathbf{y}, {{\varvec{\upbeta}}(\uptau )}^{k},{\upsigma }^{k-1}),\\ {\upsigma }^{k}\mathrm{from P }({\upsigma }^{k}| \mathbf{y},{{\varvec{\upbeta}}(\uptau )}^{k},{{\varvec{v}}}^{k})\end{array}$$

After obtaining the sample sequence in step 3, the sample sequence needs to be averaged empirically to obtain parameter estimation of **β**(τ), $v$, and $\upsigma$ [29]. Also from step 3, it is needed to check a convergence from the sample sequence that is generated from Gibbs sampling.

In this study, we used the Brq R package of MCMC with Gibbs sampling to approximate the desired country-specific marginal estimates from which posterior estimates were easily computed [31, 32]. With this regard, the Gibbs sampling algorithm was implemented with 10,000 iterations, 1,000 burn-in terms discarded, and 5 thinning intervals to make observations independent or low autocorrelation. To track the convergence of the algorithm, several diagnostic tests have been created. For this investigation, the most widely used convergence assessment methods were utilized out of a variety of testing methodologies. The three approaches trace, autocorrelation, and density plots are used in this study.

## Results

Based on the result of Table 2, among the total participants included in this study, about (76.9%) were living in rural areas. From the same result, more than half (54.7%) of maternal education was not formal education. From these households, 1,072(20.1%) and 4,251(79.9%) used improved and unimproved toilet facilities respectively. Concerning water resources, the result of this study shows that 3,272 (61.5%) and 2,051 (38.5%) households have improved and unimproved drinking water sources (Table 2). A large percentage (93.2%) of mothers were married, and more than half (54.3%) of children were ever breastfed and not currently breastfed. When we look at the number of children aged under 5 in household members, 2380 (44.7%) of them had two members and the majority (65.5%) of children had from five to nine household members (Table 2).

**Table 2:** Summary measures for a categorical sample of the socio-economic and demographic characteristics of children

**Variables**	**Frequency**	**Percentage**
Type of Toilet Facility		
Improved toilet	1072	20.1
Unimproved toilet	4251	79.9
Source of Drinking Water		
Improved water	3272	61.5
Unimproved water	2051	38.5
Type of Birth		
Singleton	5200	97.7
Multiple	123	2.3
Sex of Child		
Male	2719	51.1
Female	2604	48.9
Maternal Education		
No formal education	2914	54.7
Primary	1672	31.4
Secondary and above	737	13.8
Wealth Index		
Poorest	1806	33.9
Poorer	924	17.4
Middle	742	13.9
Richer	691	13.0
Richest	1160	21.8
Religion		
Orthodox	1518	28.5
Protestant	987	18.5
Muslim	2714	51.0
Others(_catholic, tradition, and other_)	104	2.0
Place of Residence		
Urban	1230	23.1
Rural	4093	76.9
Marital Status		
Not married	361	6.8
Married	4962	93.2
Duration of Breastfeeding		
Ever breastfed and not currently breastfed	2893	54.3
Never breastfed	225	4.2
Still breastfeeding	2205	41.4
Number of Children 5 And Under In Household		
Less than two	2023	38.0
2	2380	44.7
3 or more	920	17.3
Number of Household Members		
<=4	1454	27.3
5-9	3485	65.5
10=>	384	7.2

The median BMI has the same value as the 50^th^ percentile or the second quantile (15.32) values. The median (50^th^ percentile) maternal age of the sampled household was 28 years with a range of 15 to 49 years and also similar to current age children were 29 months with a range between 0 to 59 months (Table 3).

**Table 3:** Study result of children's BMI, current age of children, and maternal age

**Level**	**BMI**	**Current Age of children(month)**	**Maternal age**
5^th^ percentile	12.92	2	20
25^th^ percentile, the first quantile (Q_1_)	14.31	14	25
50^th^ percentile, median, or the second quantile (Q_2_)	15.32	29	28
75^th^ percentile, the third quantile (Q_3_)	16.38	44	33
85^th^ percentile	17.06	50	35
95^th^ percentile	18.27	55	40

**Fig. 1:**
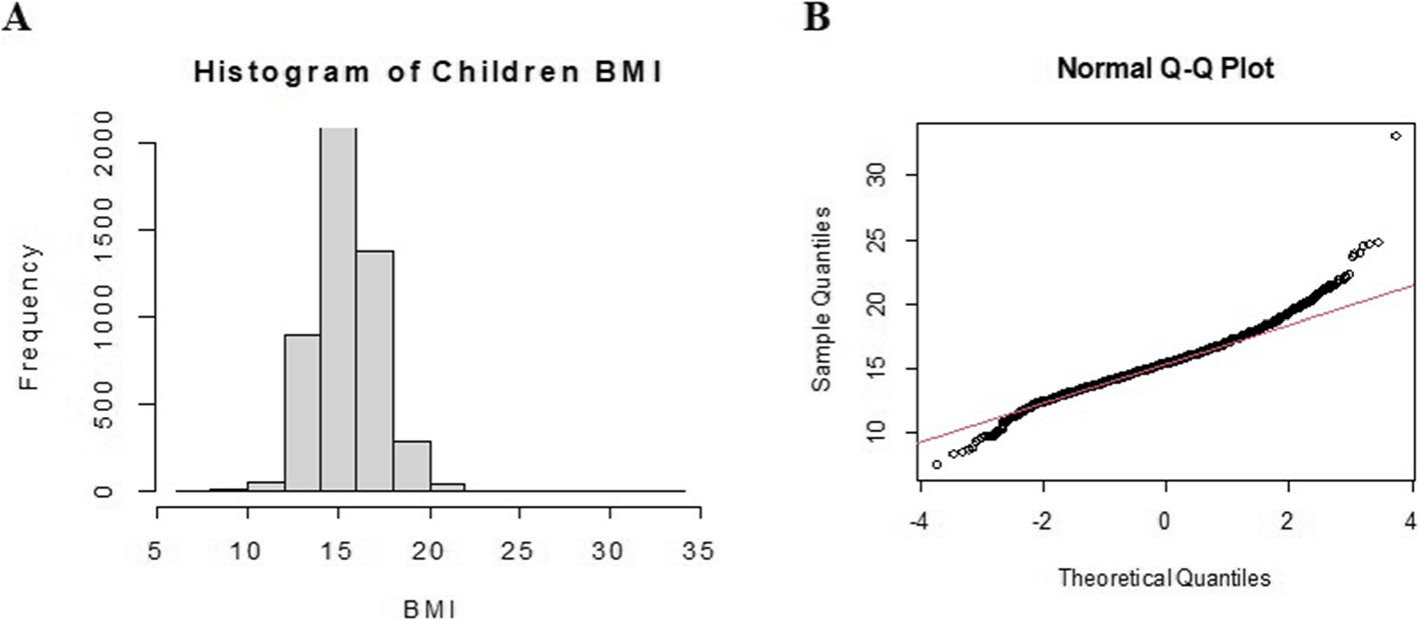
**A** Histogram and (**B**) Normal Q-Q plot for under-five children BMI data

Figure 1 (A) presents the histogram for the children's BMI. Based on the figure, it could be seen that the distribution of BMI is asymmetric, thus the distribution is not normal. Figure 1 (B) shows a normal Q-Q plot for the data. This figure also proves that the normality assumption is violated linear regression model in this children's BMI data and any outliers are in the data. To model the BMI of under-five children, the quantile regression approach was then implemented in this study.

The result from the Bayesian quantile regression model identified that the significant predictor variables at different quantile levels were presented in Table 4. At 0.05 (lower) quantile level: the results of the study showed that the current age of children, number of household members, maternal age, maternal education, religion, sex of children, region, and wealth index were found to have a significant effect on the BMI of under-five children. As the result indicated, the current age of children is negatively related to under-five children's BMI. The rate of change of the BMI of under-five children is -0.007 with a 95% credible interval (CI) = (-0.010, -0.004) at a lower quantile per unit change of current age of child keeping all the other variables constant.

**Table 4:** Parameter estimation of Bayesian quantile regression of under-five children BMI in Ethiopia

**Quantile levels**	**0.05**	**0.25**	**0.5**	**0.75**	**0.85**	**0.95**
Parameters	$\upbeta$(CI)	${\varvec{\upbeta}}$**(CI)**	${\varvec{\upbeta}}$**(CI)**	${\varvec{\upbeta}}$**(CI)**	${\varvec{\upbeta}}$**(CI)**	${\varvec{\upbeta}}$**(CI)**
Intercept	12.91 [12.57, 13.25]c^a^	14.56 [14.23, 14.92]^a^	16.00 [15.60, 16.41]^a^	17.08 [16.71, 17.46]^a^	17.66 [17.24, 18.07]^a^	19.01 [18.53, 19.49]^a^
Current Age of Children (AGE)	-0.007 [-0.01, -0.004]^a^	-0.024[-0.027,-0.02]^a^	-0.03 [-0.04, -0.02]^a^	-0.04 [-0.043 -0.03]^a^	-0.046 [-0.05, -0.04]^a^	-0.055 [-0.06, -0.05]^a^
Duration of Breastfeeding (Ref. ever and not currently breastfed)						
never breastfed (BF1)	-0.13 [-0.34, 0.12]	-0.23 [-0.43, -0.04]^a^	-0.20 [-0.38, -0.02]^a^	-0.12 [-0.30 0.05]	-0.17 [-0.36, 0.04]	0.045 [-0.21, 0.31]
still breastfeeding (BF2)	-0.01 [-0.15, 0.12]	-0.11 [-0.22, 0.01]	-0.14 [-0.26, -0.03]^a^	-0.15 [-0.27, -0.03]^a^	-0.19 [-0.32, -0.06]^a^	-0.25 [-0.41, -0.10]^a^
Number of Household Members (Ref. less than or equal to four)						
Five to nine (HS1)	-0.22 [-0.31, -0.12]^a^	-0.10 [-0.19, -0.006]^a^	-0.04 [-0.15, 0.05]	-0.06 [-0.16 0.03]	-0.044 [-0.157, 0.06]	-0.10 [-0.23, 0.02]
Greater than or equal to ten (HS2)	-0.17 [-0.37, 0.01]	-0.25 [-0.44, -0.08]^a^	-0.04 [-0.23, 0.14]	0.01 [-0.16, 0.18]	0.03 [-0.18, 0.24]	0.22 [-0.019 0.48]
Maternal Age (MA)	0.01 [0.005, 0.02]^a^	0.011 [0.005, 0.018]^a^	0.008 [0.001, 0.01]^a^	0.0086 [0.001, 0.01]^a^	0.012 [0.005, 0.02]^a^	0.02 [0.011, 0.029]^a^
Maternal Education (Ref. No formal education)						
Primary education(ME1)	0.19 [0.08, 0.29]^a^	0.03 [-0.06, 0.13]	0.08 [-0.01, 0.17]	-0.004 [-0.10, 0.09]	0.004 [-0.09, 0.10]	-0.016 [-0.15, 0.12]
Secondary and Above (ME2)	0.44 [0.29, 0.58]^a^	0.18 [0.06, 0.30]^a^	0.05 [-0.10; 0.18]	0.04 [-0.10, 0.20]	0.006 [-0.14, 0.17]	0.10 [-0.089, 0.30]
Marital Status (Ref. not married)						
Married (MS1)	-0.008 [-0.13, 0.13]	-0.11 [-0.25, 0.02]	-0.12 [-0.31, 0.04]	-0.05 [-0.19, 0.07]	0.004 [-0.17, 0.18]	-0.36 [-0.61, -0.11]^a^
Number of Children Age 5 And Under In Household (Ref. less than two )						
Two (NUFC1)	-0.07 [-0.17, 0.02]	0.07 [-0.01, 0.15]	0.05 [-0.03, 0.14]	0.10 [0.01, 0.20]^a^	0.11 (0.02, 0.21)^a^	-0.14 [-0.25, -0.03]^a^
Three or more(NUFC2)	0.13 [-0.007, 0.27]	0.05 [-0.07, 0.16]	-0.10 [-0.22, 0.02]	-0.06 [-0.19, 0.06]	-0.02 [-0.17, 0.13]	-0.065 [-0.24, 0.12]
Place of Residence (Ref. Urban)						
Rural (PA1)	0.12 [-0.01, 0.24]	0.25 [0.12, 0.37]^a^	0.21 [0.06, 0.34]^a^	0.12 [-0.02, 0.27]	0.05 [-0.10, 0.21]	0.16 [-0.0008, 0.33]
Religion (Ref. orthodox)						
Protestant (Rel1)	0.22 [0.07, 0.37]^a^	-0.03 [-0.17, 0.10]	-0.16 [-0.31, -0.02]^a^	-0.17 [-0.31, -0.02]^a^	-0.20 [-0.37, -0.04]^a^	-0.49 [-0.67, -0.32]^a^
Muslim (Rel2)	-0.01 [-0.15, 0.10]	-0.05 [-0.18, 0.06]	-0.10 [-0.23, 0.01]	-0.08 [-0.23, 0.06]	-0.14 [-0.30, -0.004]^a^	-0.11 [-0.28, 0.046]
Others (Rel3)	-0.50 [-0.84, -0.15]^a^	-0.54 [-0.86, -0.24]^a^	-0.63 [-0.91, -0.37]^a^	-0.66 [-0.98, -0.34]^a^	-0.69 [-1.06, -0.31]^a^	-0.663 [-1.07, -0.24]^a^
Sex of children (Ref. Male)						
Female (sex1)	-0.26 [-0.34, -0.18]^a^	-0.25 [-0.32, -0.17]^a^	-0.27 [-0.34, -0.19]^a^	-0.20 [-0.28, -0.13]^a^	-0.14 [-0.22, -0.06]^a^	-0.13 [-0.23, -0.03]^a^
Type of Birth (Ref. Singleton)						
Multiple (TB1)	0.01 [-0.25, 0.25]	-0.25 [-0.44, -0.04]^a^	-0.10 [-0.36, 0.15]	-0.06 [-0.34, 0.18]	-0.15 [-0.36, 0.059]	-0.34 [-0.66, 0.02]
Type of Toilet Facility (Ref. improved toilet)						
Unimproved toilet (toilet1)	0.08 [-0.02, 0.21]	0.02 [-0.09, 0.14]	-0.01 [-0.13, 0.10]	-0.0009 [-0.12, 0.12]	0.06 [-0.07, 0.20]	-0.13 [-0.311, 0.03]
Region (Ref. Tigray)						
Afar (Region2)	-0.32 [-0.57, -0.08]^a^	-0.11 [-0.33, 0.10]	-0.16 [-0.38, 0.05]	-0.05 [-0.28, 0.17]	-0.02 [-0.24, 0.21]	0.25 [-0.005, 0.50]
Amhara (Region3)	-0.13 [-0.35 0.07]	0.06 [-0.11, 0.24]	-0.04 [-0.21, 0.13]	0.08 [-0.10, 0.28]	0.065 [-0.12 0.25]	0.24 [0.05, 0.42]^a^
Oromia (Region4)	0.29 [0.08, 0.49]^a^	0.53 [0.34, 0.71]^a^	0.64 [0.44, 0.84]^a^	0.79 [0.60, 1.00]^a^	1.01 [0.82, 1.20]^a^	1.35 [1.14, 1.58]^a^
Somalia (Region5)	-0.72 [-0.96, -0.49]^a^	-0.52 [-0.74, -0.31]^a^	-0.51 [-0.75, -0.28]^a^	-0.55 [-0.79, -0.31]^a^	-0.62 [-0.83, -0.38]^a^	-0.17 [-0.43 0.07]
Benishangul (Region6)	0.02 [-0.17, 0.23]	0.11 [-0.07, 0.30]	0.01 [-0.20, 0.20]	-0.04 [-0.23, 0.16]	-0.003 [-0.22, 0.22]	0.49 [0.23, 0.75]^a^
SNNPR (Region7)	0.01 [-0.19, 0.21]	0.50 [0.31, 0.69]^a^	0.55 [0.35, 0.74]^a^	0.57 [0.37, 0.77]^a^	0.59 [0.37, 0.81]^a^	1.29 [1.02, 1.56]^a^
Gambela (Region8)	-0.48 [-0.69, -0.27]^a^	-0.17 [-0.40, 0.04]	-0.14 [-0.37, 0.09]	-0.11 [-0.32, 0.10]	-0.05 [-0.28, 0.17]	0.57 [0.30, 0.85]^a^
Harari (Region9)	0.10 [-0.12, 0.34]	0.40 [0.17, 0.62]^a^	0.41 [0.18, 0.62]^a^	0.51 [0.27, 0.76]^a^	0.61 [0.36, 0.86]^a^	1.16 [0.91, 1.40]^a^
Addis Abeba(Region10)	0.35 [0.09, 0.60]^a^	0.83 [0.61, 1.06]^a^	0.76 [0.52, 0.98]^a^	0.84 [0.58, 1.13]^a^	0.96 [0.70, 1.26]^a^	1.66 [1.38, 1.94]^a^
Dire Dawa (Region11)	-0.18 [-0.40, 0.04]	0.08 [-0.14, 0.31]	-0.03 [-0.25, 0.18]	0.08 [-0.16, 0.33]	0.10 [-0.13, 0.36]	0.44 [0.19, 0.71]^a^
Source of Drinking Water (Ref. improved water)						
Un improved water (water1)	-0.02 [-0.11, 0.06]	0.02 [-0.05, 0.10]	0.037 [-0.04, 0.11]	0.029 [-0.05, 0.12]	0.009 [-0.08, 0.10]	0.0009 [-0.10, 0.11]
Wealth Index (Ref. Poorest)						
Poorer (WI1)	0.10 [-0.03, 0.24]	0.18 [0.07, 0.29]^a^	0.104 [-0.01, 0.22]	0.07 [-0.05, 0.20]	-0.01 [-0.14 0.13]	-0.06 [-0.21, 0.09]
Middle (WI2)	0.43 [0.30, 0.56]^a^	0.35 [0.22, 0.48]^a^	0.16 [0.04, 0.29]^a^	0.08 [-0.04, 0.21]	-0.029 [-0.17, 0.12]	-0.003 [-0.18, 0.16]
Richer (WI3)	0.18 [0.04, 0.33]^a^	0.40 [0.26, 0.55]^a^	0.44 [0.29, 0.59]^a^	0.51 [0.36, 0.66]^a^	0.43 [0.26, 0.61]^a^	0.436 [0.25, 0.62]^a^
Richest (WI4)	0.37 [0.17, 0.55]^a^	0.46 [0.28, 0.63]^a^	0.38 [0.19, 0.56]^a^	0.28 [0.08, 0.48]^a^	0.29 [0.08, 0.51]^a^	0.10 [-0.11, 0.32]

^a^indicates that significant

According to the result, the female child, number of household members (five to nine), and region (Afar, Somalia, and Gambela) are negatively related to under-five children's BMI. At the lower quantile, under-five children's BMI decreased by 0.261 with CI = (-0.341, -0.181) for females as compared to male children by retaining the other factors constant. At the lower quantile, the under-five children's BMI decreased by 0.327 with CI = (-0.574, -0.088), 0.728 with CI = (-0.964, -0.499), and 0.481 with CI = (-0.690, -0.273) for children’s families lived in Afar, Somalia, and Gambela region respectively as compared to Tigray region by setting the other variables constant (Table 4).

Whereas, the findings showed that maternal age, maternal education, religion (Protestant), region (Oromia and Addis Abeba), and wealth index (middle, richer, and richest) are positively related to an under-five children's BMI. The under-five children's BMI increased by 0.012 with CI = (0.005, 0.019) for every one-unit change in the current age of the mother, holding all the other factors constant at a lower quantile level. Similarly, the under-five children's BMI increased by 0.193 with CI = (0.086, 0.292) and 0.444 with CI = (0.294, 0.582) for mothers who attend primary education and secondary and above education respectively as compared to no formal education by leaving the other variables constant at the lowest quantile level.

At 0.85 (higher) quantile, the current age of children, duration of breastfeeding, the current age of mother, number of children who are aged five and under, religion, sex of children, region, and wealth index have a significant effect on the BMI of under-five children. From this result, the current age of children is negatively related to under-five children's BMI ($\beta$ = -0.046, CI (-0.050, -0.043)). Similarly, duration of breastfed (still breastfeeding), religion, sex of a child, and region (Somalia) are negatively related to under-five children's BMI. At the higher quantile, the under-five children's BMI decreased by 0.190 in CI (-0.326, -0.060) for children still breastfeeding as compared to ever and not currently breastfeeding by setting the other factors constant.

At the (highest) 95^th^ quantile, the current age of children, duration of breastfeeding, maternal age, marital status, number of children age five and under, religion, sex of children, region, and wealth index showed a significant effect on BMI of under-five children (Table 4). The study showed that the current age of children is negatively related to under-five children's BMI. At the highest quantile level, the under-five children's BMI decreased by 0.055 within CI (-0.059, -0.050) for every one-unit change in the current age of a child, holding all the other factors constant. Furthermore, maternal age and wealth index (richer) have positively related to an under-five children's BMI. The result showed that the under-five children's BMI increased by 0.436 with CI = (0.256, 0.621) for the richer wealth index family as compared to the poorest wealth index family by holding the other factors constant (Table 4).

## Convergence checking at different quantile levels

**Fig. 2:**
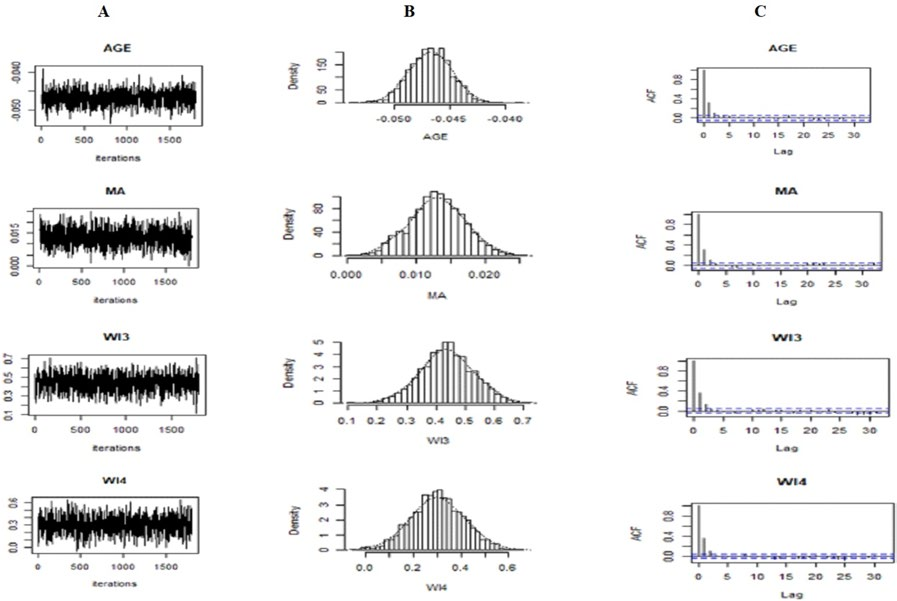
Convergence diagnosis for sample coefficients when Tau = 0.85: (**A**) trace plot, (**B**) posteriors density plot, and (**C**) autocorrelation plot. Note: Current age children = AGE, maternal age = MA, Oromia = Region4, richer = WI3, and richest =WI4

As a result, shown in the trace plots in Fig. 2A, all generated samples lie within two parallel horizontal lines, straight lines that did not show up and down periods, centered at respective values, and no trends are detected. For all simulated parameters, the trace plot indicates a good convergence since the independently generated chains are mixed or overlapped. The marginal posterior density plots in Fig. 2B below inform us that the conditional posterior distributions are the desired stationary univariate normal. This shows that all posterior estimates converged.

The finding of the study shows that Fig. 2C indicates that the decrease in the empirical autocorrelation of posterior samples proves that the underlying chains are stationary. The given below independently generated chains demonstrated good chain mixture, an indication of convergence. This shows that all posterior estimates converged. Not all trace, density, and autocorrelation plots are presented here; the remaining plots can be the same as like to this. The results obtained from these convergence diagnostics indicate that our algorithm used in the Bayesian quantile regression approach could produce adequate and acceptable values of the estimated parameter.

## Discussion

Based on the findings of the study using the 2019 mini EDHS data, several variables were identified connected to various quantiles of BMI in children under the age of five. One notable factor that was found to lower under-five children's BMI in both the higher and lower quantile levels was their current age. These findings highlight the importance of age-specific interventions that target different age groups of children under the age of five. Such interventions can focus on providing appropriate nutrition, dietary counseling, and health education tailored to the specific needs of children at different stages of development. This result is consistent with previous studies conducted in Ethiopia [11], Sudan [24], and China [33] which found age to be an important factor influencing children's BMI. The study found that breastfeeding has a negative association with under-five child BMI in the upper quantile. This suggests that breastfeeding helps prevent excessive weight gain and reduces the likelihood of children becoming overweight. This finding is consistent with the findings of other studies conducted in China [34] and Greece [35]. These findings emphasize the significance of exclusive breastfeeding and encouraging mothers to exclusively breastfeed their infants for the first six months and continue breastfeeding alongside appropriate complementary feeding practices can contribute to the healthy growth and development of children.

The finding of this study also showed that maternal age is positively related to the BMI of under-five children in the upper quantile level. Younger mothers may engage in more physical activities, provide active stimulation, and promote healthy eating habits, resulting in lower BMI levels for their children. This suggests that younger mothers have more energy and are better able to actively care for their children and provide better care and nutrition for their children, leading to healthier weights. This finding was in agreement with another study conducted in Ethiopia [36]. But this result contradicted study findings conducted in Ethiopia [37]. This may be attributed to the majority of children whose mother was young and adult in this study, which leads to a healthy weight.

Our findings also showed that one of the most important factors affecting under-five children's BMI at different quantiles was the sex of a child. Female children have a worse relationship with BMI at both the lower and upper quantiles for children under five than male children. Female children have a worse relationship with BMI at both the lower and upper quantiles compared to male children, suggesting that there may be gender-related differences in factors influencing BMI in early childhood. This finding is consistent with previous studies conducted in Ethiopia [11] and Sudan [24]. The finding of the study has also shown that a mother’s education significantly affects under-five children’s BMI in the lower quantile level. Children whose mothers attended primary education level had a positive association with under-five child BMI, while children whose mothers had no formal education had a negative association. This indicates that education enables mothers to implement basic health knowledge effectively. It also enhances their ability to navigate healthcare facilities, interact with healthcare professionals, adhere to treatment recommendations, and maintain a clean environment for their children. This finding is in line with the study findings conducted in Taiwan [38] and Ethiopia [39, 40], indicating that maternal education plays a crucial role in shaping children's BMI outcomes.

The findings of this study indicate that religion significantly influences under-five children's BMI at various quantiles. Specifically, families with children who practice the Protestant religion have a more favorable relationship between their children's BMI under the age of five, particularly in the lower quantiles.

Moreover, this findings of this study showed that religion significantly influences under-five children's BMI at various quantiles. According to the findings, families with children who practice the protestant religion have a favourable relationship between their children's BMI under the age of five and those who practice the Orthodox religion in the lower quintile. This finding is consistent with other studies conducted in Ethiopia [41]. However, a previous study conducted in Ethiopia [11] did not find a significant association between under-five children's BMI and religion. The variation in findings could be attributed to several factors. Firstly, different statistical models, such as the Bayesian quantile regression model used in this study, may yield different results. Secondly, the majority of children from families practicing the Protestant religion in this study came from households with better wealth indexes and educated mothers. These socio-economic and educational factors could have influenced the relationship between religion and children's BMI.

Similarly, it was discovered that geography had an impact on under-five children's BMI at various quantiles. According to our findings, a child who lives in Afar, Somalia, and Gambela regions has a worse relationship with their under-five child's BMI than a child who lives in the region of Tigray in the lower quantile. On the other hand, children living in the Amhara, Oromia, Benishangul, SNNPR, Gambela, Harari, Addis Abeba, and Dire Dawa regions have a more favorable relationship with under-five children's BMI compared to those in the Tigray region in the upper quantiles. This result is also consistent with the finding of a study in Ethiopia [11], suggesting that geography plays a role in children's BMI outcomes. The varied associations between geography and under-five children's BMI in different quantiles may reflect regional differences in factors such as access to healthcare, socio-economic conditions, cultural practices, and dietary patterns.

The BMI of under-five children at various quantiles was found to be significantly influenced by the home wealth index. In contrast to poorer wealth index families in the lower quantile level and the upper quantile level, the study's findings on the wealth index of family richer and richest wealth index families were positively related to under-five child BMI. This finding is consistent with previous research studies conducted in Ethiopia [39, 42] and also the study results in Kenya [43].

The possible reason may be, that families with higher wealth index often have greater access to resources such as nutritious food, and a more favorable living environment. These factors may contribute to increasing BMI for under five children. Furthermore, the middle-level wealth index of the family is positively related to under-five child BMI as compared to poorer wealth index families in the lower quantile. This result also seems to agree with the previous finding of the study in Bangladesh [44], further supporting the notion that a moderate level of wealth can still have a positive impact on children's BMI, relative to families with lower wealth index.

## Limitations of the study

This study had certain limitations, one of which was the unavailability of variables such as maternal BMI and children's weight at birth in the mini EDHS data set. This may have an impact on the result of the study.

## Conclusions

The study findings indicate that several factors have a significant effect on under-five child BMI at both lower and upper quantile levels. The study also showed that the BMI of children under the age of five in Ethiopia is significantly influenced by socioeconomic, behavioral, and demographic factors. The results revealed that the present age of the children, the sex of the children, the age of the mothers, the family's religion, the location, and the wealth index all had a significant impact on the BMI of under-five children at both the lower and upper quantile levels. Additionally, it was discovered that mothers' education levels had a substantial impact on the BMI of under-five children in lower quantile levels.

Thus, we recommend that the education sector should promote maternal education and policies to reduce cultural and gender barriers. Further research is needed to establish the causal relationships between the identified factors and under-five children's BMI in Ethiopia. This would provide a deeper understanding of the factors influencing BMI and inform more targeted interventions and policies to improve the nutritional status of young children in the country.

## Data Availability

The dataset used and analyzed during the current study is openly available from EDHS website (https://dhsprogram.com).

## Abbreviations

ALD: Asymmetric Laplace Distribution
BMI: Body Mass Index
Brq: Bayesian Quantile Regression
CI: Credible Interval
EDHS: Ethiopian Demographic and Health Survey
MCMC: Markov Chain Monte Carlo
pdf: Probability Density Function
QRM: Quantile regression models
SNNPR: South Nations Nationalities and Peoples Representative
